# Development of an Automated MRI-Based Diagnostic Protocol for Amyotrophic Lateral Sclerosis Using Disease-Specific Pathognomonic Features: A Quantitative Disease-State Classification Study

**DOI:** 10.1371/journal.pone.0167331

**Published:** 2016-12-01

**Authors:** Christina Schuster, Orla Hardiman, Peter Bede

**Affiliations:** Quantitative Neuroimaging Group, Academic Unit of Neurology, Biomedical Sciences Institute, Trinity College Dublin, Ireland; Banner Alzheimer's Institute, UNITED STATES

## Abstract

**Background:**

Despite significant advances in quantitative neuroimaging, the diagnosis of ALS remains clinical and MRI-based biomarkers are not currently used to aid the diagnosis. The objective of this study is to develop a robust, disease-specific, multimodal classification protocol and validate its diagnostic accuracy in independent, early-stage and follow-up data sets.

**Methods:**

147 participants (81 ALS patients and 66 healthy controls) were divided into a training sample and a validation sample. Patients in the validation sample underwent follow-up imaging longitudinally. After removing age-related variability, indices of grey and white matter integrity in ALS-specific pathognomonic brain regions were included in a cross-validated binary logistic regression model to determine the probability of individual scans indicating ALS. The following anatomical regions were assessed for diagnostic classification: average grey matter density of the left and right precentral gyrus, the average fractional anisotropy and radial diffusivity of the left and right superior corona radiata, inferior corona radiata, internal capsule, mesencephalic crus of the cerebral peduncles, pontine segment of the corticospinal tract, and the average diffusivity values of the genu, corpus and splenium of the corpus callosum.

**Results:**

Using a 50% probability cut-off value of suffering from ALS, the model was able to discriminate ALS patients and HC with good sensitivity (80.0%) and moderate accuracy (70.0%) in the training sample and superior sensitivity (85.7%) and accuracy (78.4%) in the independent validation sample.

**Conclusions:**

This diagnostic classification study endeavours to advance ALS biomarker research towards pragmatic clinical applications by providing an approach of automated individual-data interpretation based on group-level observations.

## Introduction

Amyotrophic Lateral Sclerosis (ALS) is a relentlessly progressive neurodegenerative condition. The average diagnostic delay from symptom onset to definite diagnosis is 12 months, which not only delays neuroprotective treatment, recruitment to pharmaceutical trials, multidisciplinary interventions and care planning, but misdiagnosis of ALS to other conditions may lead to unnecessary interventions [1, 2]. Factors contributing to diagnostic delay in ALS include disease heterogeneity, insidious symptom onset, and presentation with non-motor symptoms.

While research protocols can reliably capture genotype, and phenotype-specific changes in ALS, [3] the role of magnetic resonance imaging in clinical practice remains limited to the exclusion of intracranial and spinal pathology which may mimic ALS. Contrary to the qualitative approach used in clinical radiology, the majority of quantitative ALS studies are based on group-level analyses, and rely either on comparative interpretation or correlations with clinical variables. [4–6] In recent years however, there has been an increased interest in the diagnostic classification of individual MRI data sets.

Machine-learning and support vector machine classifier-analyses [7, 8] have been increasingly applied to neurodegenerative conditions [9] including ALS. [10] Reports on the diagnostic sensitivity of diffusion-tensor imaging (DTI) alone are inconsistent. A meta-analysis of 11 DTI studies, including 221 ALS patients and 187 healthy control subjects, suggested that the diagnostic accuracy of corticospinal tract DTI alone may be insufficient. [11] Smaller studies on the other hand, reported relatively good specificity and sensitivity based on the discriminant analyses of diffusivity measures.[12] Functional magnetic resonance imaging studies in ALS achieved over 71% diagnostic accuracy using a support-vector machine approach. [10] With few exceptions, [13] the commonest shortcomings of these studies include reliance on a single imaging measure, evaluation of a single anatomical structure and a categorical classification outcome instead of probability values. Additionally, classification studies often restrict their discriminating features to significant voxels only, rendering their model sample-specific i.e. overfitting and hindering model generalisability. Moreover, classification models are seldom cross-validated in an independent sample.

Based-on the available literature, we hypothesised that the diagnostic accuracy of a classification models may be increased by incorporating multiple imaging indices of multiple disease-defining anatomical structures, and that instead of binary categorical classification, it is feasible to provide a diagnostic probability scores. Accordingly, the objective of this study was to develop a robust, imaging based automatic diagnostic protocol, which determines the probability of a single MRI data set representing changes consistent with ALS.

## Methods

All participants provided written, informed consent in accordance with the Medical Ethics Approval of the research project (Ethics (Medical Research) Committee—Beaumont Hospital, Dublin, Ireland).

### Overview

**Fig 1:**
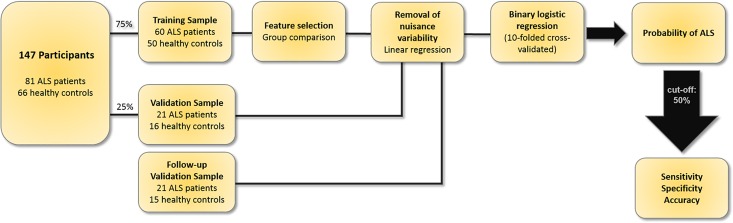
The flowchart of the development and evaluation of the diagnostic classification protocol.

Imaging data were divided into a “*training sample*” to develop the probability algorithm, and a “*validation sample*” to assess its generalisability. (**Fig 1**) Discriminating input features were selected based on group comparisons between patients and controls in the training sample. The selected features were adjusted for age-related differences [14]. A binary logistic regression analysis was then conducted. The resulting algorithm was validated in the independent “*validation sample*” and further assessed based on follow-up scans of the same participants. The sensitivity, specificity and accuracy of this approach were evaluated in each sub-cohort separately.

### Participants

This study incorporates imaging data from 147 participants; 81 patients with ALS and 66 healthy controls (HC). All participating patients had probable or definite ALS according to the revised El Escorial criteria. [15] Patients with a co-morbid diagnosis of frontotemporal dementia according to the Rascovsky Criteria were excluded because of the confounding effects of imaging changes associated with this phenotype. [16, 17] 75% of the data were randomly allocated to the “*training sample*” and 25% to the “*validation sample*”. Participants of the “*validation sample*” were rescanned longitudinally to assess the effect of disease duration of diagnostic accuracy. We refer to this cohort as “*follow-up validation sample*”. The demographic profile of each cohort is presented in **Table 1.**

**Table 1:** Demographic and clinical data of participants.

	Training Sample			Validation Sample			Follow-up Validation Sample		
	ALS	HC	p-values	ALS	HC	p-values	ALS	HC	p-values
n	60	50		21	16		21	15	
Male sex, n	39	24	p = .1	10	6	p = .74	12	9	p = .86
Age, y(mean/SD)	59.9(10.88)	60.6 (8.8)	p = .68	62.5 (10.5)	60.6 (9.4)	p = .56	62.9 (10.4)	61.6 (9.2)	p = .69
Handedness(right/left)	52/ 8	47/ 3	p = .33	19 /2	14/ 2	p = .77	19/2	13/2	p = .72
Disease Duration months (mean/SD)*	26.1(19.5)			20.6 (15.9)			25.8 (15.6)		
Site of onset(bulbar/spinal/ respiratory)	19/ 40/1			6/15/0			6/15/0		
ALSFRS-R(mean, SD)	38.2 (6.2)			39 (7)			35.3(8.2)		
Interval between scans months (mean/ SD)							5.3 (1.5)	4.2 (0.8)	p< .05

* Disease duration from symptom onset until date of scan

### Imaging data acquisition

MR data were acquired on a 3 Tesla Philips Achieva system with a gradient strength of 80 mT/m and slew rate of 100 T/m/s using an 8-channel receive-only head coil. T1-weighted images were obtained using a three-dimensional inversion recovery prepared spoiled gradient recalled echo (*IR-SPGR*) sequence with FOV = 256×256×160 mm, spatial resolution = 1 mm3, TR/TE = 8.5/3.9 ms,TI = 1060 ms, flip angle = 8°, SENSE factor = 1.5. DTI images were acquired using a spin-echo planar imaging (SE-EPI) sequence with a 32-direction Stejskal-Tanner diffusion encoding scheme: FOV = 245 x 245 x 150 mm, spatial resolution = .5 mm3, 60 slices with no interslice gap, TR/TE = 7639 / 59 ms, SENSE factor = 2.5, b-values = 0, 1100 s/mm2, with SPIR fat suppression and dynamic stabilisation in an acquisition time of 5 min 41 s.

### Imaging data analysis

#### Training sample

Discriminatory features were selected based on comparative analyses in the training sample. Raw imaging data were pre-processed (described below) and comparisons between patients and controls were adjusted for age, gender and disease duration to identify brain regions affected early during the course of the disease independently of age and gender. [18] The affected brain regions identified by these analyses were anatomically segmented and imaging measures from these masks were retrieved to serve as input data for the classification model.

#### Grey matter (GM) analyses

**Fig 2:**
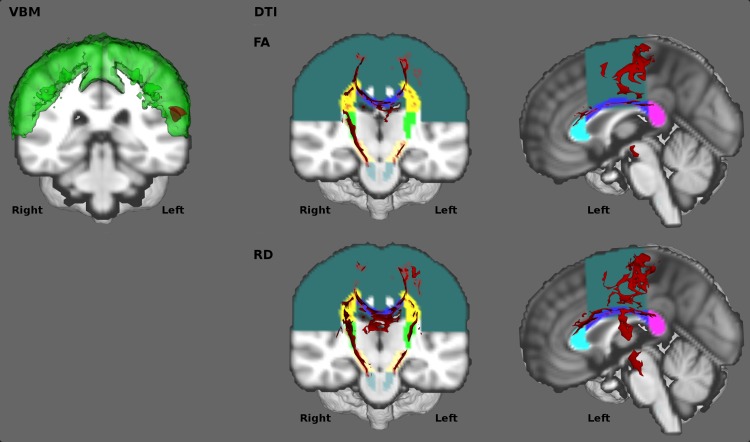
Feature selection. Left (VBM): Grey matter. Affected cortical regions at p<0.15 FWE are displayed in red, and the corresponding anatomical label, the precentral gyrus is shown in green. Middle and Right (DTI): White matter. Factional anisotropy (FA—Top) and Radial diffusivity (RD–Bottom) group comparison results are shown in red at p<0.01 FEW-TFCE. The corresponding anatomical labels colour coded as follows: dark green—lateral fibres of the corona radiata, blue—body of the corpus callosum, turquoise—genu of the corpus callosum, purple–splenium of the corpus callosum, bright yellow–inferior corona radiata, green—internal capsule, beige—mesencephalic cruri of the cerebral peduncles, grey–inferior corticospinal tracts in the pons.

A voxel-based morphometry type analysis was carried out using FSL. [19] Images were brain-extracted, tissue-types were segmented and aligned to the Montreal Neurological Institute 152 standard space using non-linear registration. A study-specific template was created including 16 randomly selected ALS patients and 16 age- and gender- matched healthy controls (ALS: male = 8, mean age = 62.9 years ± 9.3; HC: male = 8, mean age = 62.3 years ± 9.3; p = .85). All native grey matter images were then non-linearly registered to the study-specific template and modulated to correct for focal contractions and enlargement due to the non-linear component of the spatial transformation. An isotropic Gaussian kernel (σ = 3 mm) was used to smooth the modulated grey matter images. Voxel-wise generalised linear models and permutation-based non-parametric testing (10,000 permutations) were applied to determine differences between ALS patients and healthy controls using age, gender and disease duration as covariates. A trend of difference was identified in the precentral gyrus between patients and controls based on the Harvard-Oxford atlas [20] at p < .15, corrected for multiple comparisons using family-wise error (FWE). **Fig 2**The grey matter feature of the classification model was defined based on the location of these discriminatory voxels. As laterality and focality of motor cortex pathology defines motor disability in ALS [4, 21], the precentral gyrus mask of the Harvard-Oxford atlas [20] was split into left and right hemispheric regions and fed into the classification model separately.

#### White matter (WM) analyses

Diffusion weighted image pre-processing included eddy current corrections, motion corrections, and brain-tissue extraction in FSL. [22] A diffusion tensor model was fitted at each voxel, generating maps of fractional anisotropy (FA), mean diffusivity (MD), axial diffusivity (AD), and radial diffusivity (RD). Each dataset was aligned to the FMRIB58_FA standard space image. Each subject's aligned FA data was then projected onto the mean FMRIB58_FA skeleton representing the common white matter tracts and the resulting data were subsequently fed into voxel-wise cross-subject statistics. ALS patients were compared with healthy controls controlling for age, gender and disease duration, using a voxel-based generalised linear model and permutation-based non-parametric testing with 10,000 permutations. The threshold-free cluster enhancement (TFCE) method [23] was applied and the significance level was set at p < .01, corrected for multiple comparisons using family-wise error (FWE) method. Significant group differences were identified in the corpus callosum and along the corticospinal tracts in FA and RD at p < .01 (FWE), (**Fig 2**) but not in MD or AD.

Analogous to the GM analyses, discriminatory “features” were defined as the anatomical regions which included statistically significant voxels in the patients versus controls contrasts. The FSL JHU atlas was used for spatial segmentation, which consists of 48 white matter tracts labels created based on the diffusion tensor maps from 81 subjects. [24] The region of interest map for the corona radiata was manually created.

Based on the above comparative analyses, the following white matter structures were selected as input features for the classification algorithm (**Fig 2**): the genu (gCC), the body (bCC) and the splenium (sCC) of the corpus callosum, the inferior corticospinal tracts (iCST) in the pons, the mesencephalic cruri (ME) of the cerebral peduncles, the internal capsule (IC), the inferior corona radiata (iCR) and the superior corona radiata (sCR). With the exception of the midline corpus callosum segments, the left and right hemispheric segments of the corticospinal tracts were included separately, resulting in 26 white matter features in total: FA and RD of the gCC, bCC, sCC, left iCST, right iCST, left ME, right ME, left IC, right IC, left iCR, right iCR, left sCR, right sCR.

#### Validation Sample

The pre-processing steps of the “*independent validation sample*” were analogous to the pre-processing pipeline of the “*training sample*”. For grey matter analyses, data from each subject was co-registered to the template of the “*training sample*”. The same smoothing kernel was applied (σ = 3 mm) and the “feature” masks described above were used to extract average grey matter density values from the left and right precentral gyrus.

For white matter analyses, the data from each subject was co-registered to the FMRIB58a_FA standard space image. The above described gCC, bCC, sCC, left iCST, right iCST, left ME, right ME, left IC, right IC, left iCR, right iCR, left sCR, right sCR white matter masks were used to extract average FA and average RD values from each pathognomonic white matter region.

#### Removal of nuisance variability

To account for age-related differences, a linear regression model was fitted to the values of each feature in the control group using age as an independent variable. In order to prevent the removal of disease-specific changes, only control data was used. This approach has been previously described to increase classification accuracy. [14, 25]

#### Binary logistic regression

A binary logistic ridge regression model was utilised based on all age-corrected ALS-specific features and designating group as the outcome variable. The statistical software R and the package ‘glmnet’ (α = 0) [26] was utilised to carry out the logistic regression. The tuning parameter λ was selected based on ten-folded cross-validation which was repeated 100 times. The hyperparameter lambda was determined only using the training data. The model with the smallest misclassification error averaged over the 100 estimations was selected.

## Results

### Binary logistic regression

**Fig 3:**
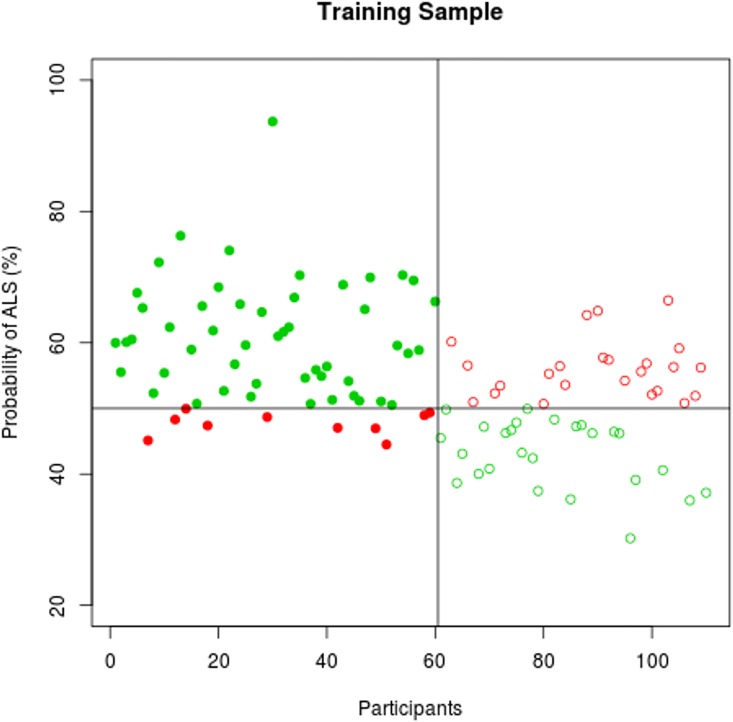
Classification accuracy in the training sample. The probability of individual participant’s MRI data demonstrating ALS-specific change based on the classification algorithm. Patients with ALS are represented by filled circles, healthy controls by empty circles. Misclassified participants are displayed in red; correctly classified participants in green.

**Fig 4:**
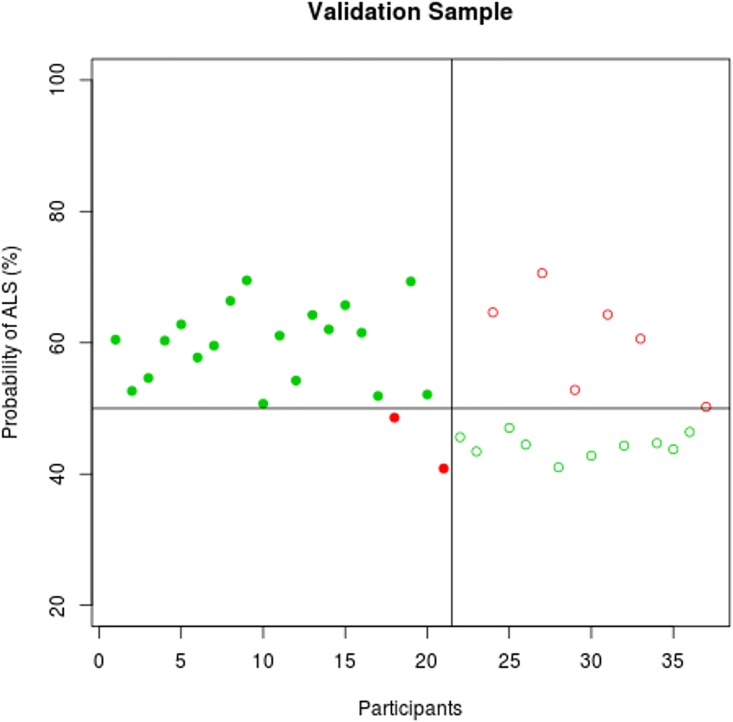
Classification accuracy in the validation sample. The probability of individual participant’s MRI data demonstrating ALS-specific change based on the classification algorithm. Patients with ALS are represented by filled circles, healthy controls by empty circles. Misclassified participants are displayed in red; correctly classified participants in green.

**Fig 5:**
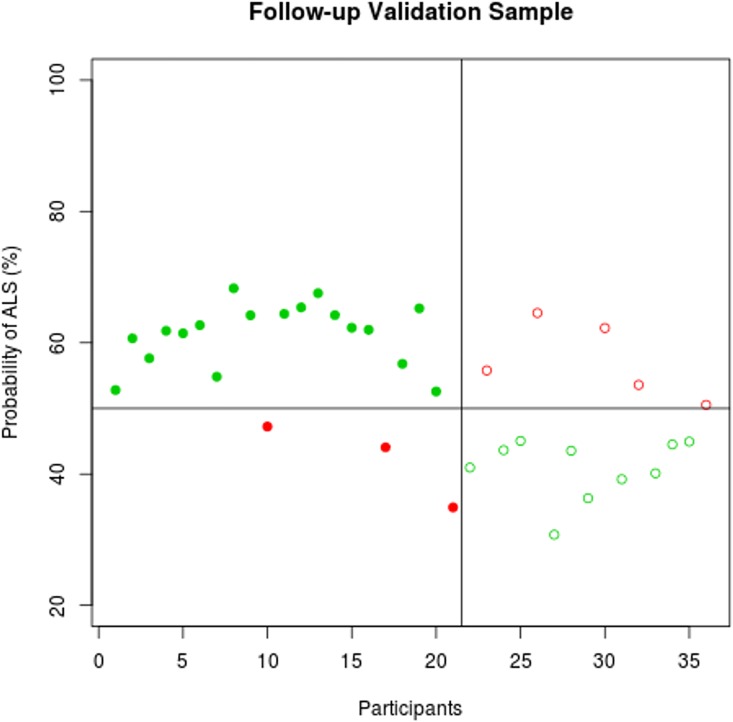
Classification accuracy in the follow-up validation sample. The probability of individual participant’s MRI data demonstrating ALS-specific change based on the classification algorithm. Patients with ALS are represented by filled circles, healthy controls by empty circles. Misclassified participants are displayed in red; correctly classified participants in green.

The predicted probability profile of individual participants to exhibit ALS-specific changes is displayed in **Figs 3–5**. The classification results in the training and the validation samples are shown in **Table 2**using a probability cut-off of 50%.

**Table 2:** Classification results using the 50% probability threshold.

	**Training Sample**			
class	ALS	HC		
Predicted class			Sensitivity	83.34%
ALS	50	24	Specificity	52.00%
HC	10	26	Accuracy	69.09%
	**Validation Sample**			
class	ALS	HC		
Predicted class			Sensitivity	90.47%
ALS	19	6	Specificity	62.50%
HC	2	10	Accuracy	78.37%
	**Follow-up Validation Sample**			
class	ALS	HC		
Predicted class			Sensitivity	85.71%
ALS	18	5	Specificity	66.67%
HC	3	10	Accuracy	77.77%

### Misclassification

The demographic profiles of misclassified individuals in the “*Training Sample*” and “*Validation sample*” are presented in **Table 3**, **Table 4**, and **Table 5**. The same patients and controls were misclassified in the “Validation sample” and in the follow-up “*validation sample*”.

**Table 3:** Comparison of correctly classified and misclassified ALS patients and controls in the training sample.

**ALS Patients**			
	True Positive	False Negative	p-values
N	50	10	
Male sex, n	32	7	P = .72
Age, y (mean, SD)	60.66 (10.46)	56.04 (12.69)	P = .30
Handedness (right/left)	44/ 6	8/ 2	P = .86
Disease duration from symptom onset until date of scan, m (mean, SD)	25.72 (16.07)	28.2 (33.01)	P = .82
Type of onset, (bulbar/ spinal/respiratory)	18/ 31/ 1	1/ 9	P = .23
ALS-FRS-R (mean, SD)	37.46 (6.57)	42 (4.06)	P < .01
Probability of ALS (mean, SD)	61.16 (8.44)	47.63 (1.79)	P < .01
**Healthy controls**			
	True Negative	False Positive	p-values
N	26	24	
Male sex, n	16	8	P = .08
Age, y (mean, SD)	60.34 (8.48)	60.99 (9.33)	P = .80
Handedness (right/left)	25/ 1	22/ 2	P = .94
Probability of ALS (mean, SD)	56.08 (4.39)	43.08 (5.07)	P < .01

**Table 4:** Comparison of correctly classified and misclassified ALS patients and controls in the validation sample.

**ALS Patients**			
	True Positive	False Negative	p-values
N	19	2	
Male sex, n	11	1	P = .83
Age, y (mean, SD)	62.96 (10.9)	57.85 (1.91)	P = .10
Handedness (right/left)	17/ 2	2/ 0	P = .63
Disease duration from symptom onset until date of scan, m (mean, SD)	21.58 (16.47)	11.5 (4.95)	P = .12
Type of onset, (bulbar/ spinal/respiratory)	6/ 13	0/ 2	P = .91
ALS-FRS-R (mean, SD)	38.58 (7.27)	43 (1.41)	P < .05
Probability of ALS (mean, SD)	59.84 (5.87)	44.73 (5.48)	P = .13
**Healthy controls**			
	True Negative	False Positive	p-values
N	10	6	
Male sex, n	7	3	P = .42
Age, y (mean, SD)	57.44 (10.07)	65.8 (5.29)	P < .05
Handedness (right/left)	9/ 1	5/ 1	P = .70
Probability of ALS (mean, SD)	44.36 (1.76)	60.53 (7.72)	P < .01

**Table 5:** Comparison of correctly classified and misclassified ALS patients and controls in the follow-up validation sample.

**ALS Patients**			
	True Positive	False Negative	p-values
N	18	3	
Male sex, n	10	2	P = .72
Age, y (mean, SD)	63.44 (11.04)	56.67 (0.38)	P < .05
Handedness (right/left)	17/ 1	2/ 1	P = .65
Disease duration from symptom onset until date of scan, m (mean, SD)	22.11 (16.58)	11.67 (8.14)	P = .14
Type of onset, (bulbar/ spinal/respiratory)	5/ 13	1/ 2	P = .84
ALS-FRS-R (mean, SD)	38.58 (7.27)	43 (1.41)	P < .05
Probability of ALS (mean, SD)	61.38 (4.69)	42.08 (6.39)	P < .05
**Healthy controls**			
	True Negative	False Positive	p-values
N	10	5	
Male sex, n	7	2	P = .58
Age, y (mean, SD)	59.35 (10.16)	65.28 (5.74)	P = .17
Handedness (right/left)	9/ 1	4/ 1	P = .59
Probability of ALS (mean, SD)	40.91 (4.57)	57.34 (5.89)	P < .01

## Discussion

Classification methods and imaging biomarkers are increasingly used in medicine, and are particularly well integrated into clinical decisions in oncology and Alzheimer’s disease. Biomarker development in ALS yielded mostly to descriptive results to date, but the establishment of multi-centre data repositories creates a unique opportunity to test classification models in cross-platform data sets. The main objective of this study was to develop a computer-aided diagnostic tool based on disease-specific pathological signatures and multiple imaging measures, which provides a diagnostic probability score.

Based on the outlined multi-modal neuroimaging approach, diagnostic classification accuracy was achieved with good sensitivity and moderate specificity. Previous classification studies of ALS did not use an independent validation sample, but relied solely on cross-validation within the training sample [11–13, 27–30] Validation in an independent patient cohort is essential to demonstrate the generalisability of the model, and it also paves the way for inclusion of cross-platform data sets. [31] While previous classification studies have relied on the highly discriminatory voxels of the initial comparisons, [13] we used anatomical labels around these regions to avoid model overfitting. This circumvents relying on maps which are uniquely specific to the training sample. Furthermore, we selected brain regions i.e.: discriminatory anatomical “features” which are affected early in the course of ALS by regressing out the effect of disease duration. As disease duration is also a key factor in classification accuracy, we evaluated diagnostic accuracy longitudinally in the independent validation cohort.

In a clinical setting, where a possible diagnosis of ALS is suspected, not only high sensitivity values and quantitative probability outcomes are desirable, but high specificity is also paramount to prevent patients with mimic neurodegenerative conditions to be misclassified as ALS. A number of mathematical classification models have been previously applied to imaging data sets, such as random forest approaches, discriminant function analysis, Naïve Bayes classification and support vector machines [32]. The advantage of the binary logistic regression model is that it provides probability outcomes, as opposed to categorical classification. Quantitative diagnostic probability outcomes are more easily integrated in clinical decision making alongside the gold standard assessments, such as the neurological examination and electrophysiological testing.

The presented study is not without limitations. The study uses a standard single-platform, single-centre approach and is not validated on data acquired from other centres. Despite advances in cross-centre harmonisation, [31] the effect of pulse sequence differences on spatial statistics is well established. [33] Multicentre MR studies have been successfully conducted in Alzheimer’s disease, [34] and the cross platform calibration of the Alzheimer’s Disease Neuroimaging Initiative (ADNI) [35] using travelling MRI phantoms has been comprehensively described. Despite the relatively high accuracy of our algorithm, our analysis of misclassifications suggests that young ALS patients, patients with high ALSFRS-r and short disease duration and older controls are the most likely to be misclassified. We acknowledge that misclassification of patients high ALSFRS-r may have implications to establishing early-stage diagnosis.

The Neuroimaging Society in ALS (NiSALS) has established a large data repository which is an ideal resource to test classification models in ALS. [36] Our study is limited to patients with classical ALS and controls. Patients with comorbid frontotemporal dementia and mimic conditions were not included. Notwithstanding the relatively modest specificity results, our data suggest that the inclusion of additional pathognomonic regions such as basal ganglia [37, 38] spinal cord [39, 40], or cerebellar measures [41] may increase the diagnostic accuracy of the model further. Moreover, the inclusion of other imaging parameters such as cortical thickness measurements, volumetrics, connectivity measures, or spectroscopy may further enhance diagnostic models [42]. From a clinical perspective, urgent work is required to develop classification models which can reliably identify early-stage ALS and distinguish it from mimic conditions and other neurodegenerative conditions. Such models also have to include anatomical regions which are not typically affected in ALS but are implicated in other neurodegenerative conditions. [43] The accurate classification of overlap syndromes such as ALS-FTD may be particularly challenging. These conditions have a less distinctive imaging signature with features of both ALS and FTD. The sensitivity of proposed classification models should be tested on presymptomatic cohorts which are likely to exhibit disease-specific imaging traits long before the manifestation of the disease. [44]

The classification methodology outlined in this study can be used beyond the initial diagnosis to segregate ALS phenotypes. ALS is an outstandingly heterogeneous condition encompassing distinctive motor phenotypes, genotypes, [45] cognitive cohorts, [46] slow and fast progressors. [45, 47] As these phenotypes have distinguishing imaging features, newly diagnosed or suspected patients could potentially be sub-phenotyped for stratification into pharmaceutical trials. Cross-sectional MRI data have been previously evaluated for their predictive value of clinical decline, [48] but have not been used to segregate patients with slow and fast progression rates. Finally, classification pipelines developed for ALS are transferable to other neurodegenerative conditions where pathological change also occurs in a unique, disease-specific anatomical pattern.

## Conclusions

The classification approach outlined in this study relies on assessing multiple imaging measures in multiple disease-defining anatomical regions in individual data sets to provide a diagnostic probability score. In an era where cross-platform harmonisation is gaining increasing momentum and acquisition protocols constantly improve, the presented approach is likely to lead to increasingly accurate diagnostic classification. Ultimately, imaging biomarkers in ALS are gradually expanding beyond their descriptive role to be developed into viable diagnostic and prognostic markers.
